# Developing a method for measurement of dehydrogenase activity in biological wastewater treatment processes applied for toxic compounds degradation

**DOI:** 10.1016/j.mex.2020.100970

**Published:** 2020-06-20

**Authors:** Mojtaba Pourakbar, Ali Behnami, Mostafa Mahdavianpour, Fatemeh Shokri Dariyan, Ehsan Aghayani

**Affiliations:** a Department of Environmental Health Engineering, Maragheh University of Medical Sciences, Maragheh, Iran; b Health and Environment Research Center, Tabriz University of Medical Sciences, Tabriz 5166614711, Iran; c Department of Environmental Health Engineering, Abadan University of Medical Sciences, Abadan, Iran; d Department of Environmental Health Engineering, School of Public Health, Shahid Beheshti University of Medical Sciences, Tehran, Iran

**Keywords:** Biomass, Enzymatic degradation, DHA

## Abstract

Biological wastewater treatment processes are among the environmentally friendly techniques for degradation of organic compounds. They are also preferred to the physical and chemical processes which are due to the ability of biological processes to treat wide range of organic compounds with lower operational costs. However, biological processes are usually affected by variation in the inlet wastewater quality and quantity. In order to investigate the performance of the wastewater treatment plant, various parameters in case of effluent quality such as COD, BOD, TSS, TDS etc. are required to be measured. Microorganisms in bioreactors use various enzymes to degrade the organic contaminants. Higher toxic organic load on the biological process may lead to the deterioration of the process performance which is due to the reduction in microbial activity of the biomass. Dehydrogenase enzyme produced in biological processes could be used as an indicator for the biological wastewater treatment. Present study introduces a simple and modified method for evaluation of biological wastewater treatment process measuring dehydrogenase activity. In the present study, the effective parameters such as incubation time and types of solvent were investigated and the best procedure is developed for measuring the dehydrogenase activity in biological wastewater treatment process.

## Graphical abstract

**Figure:**
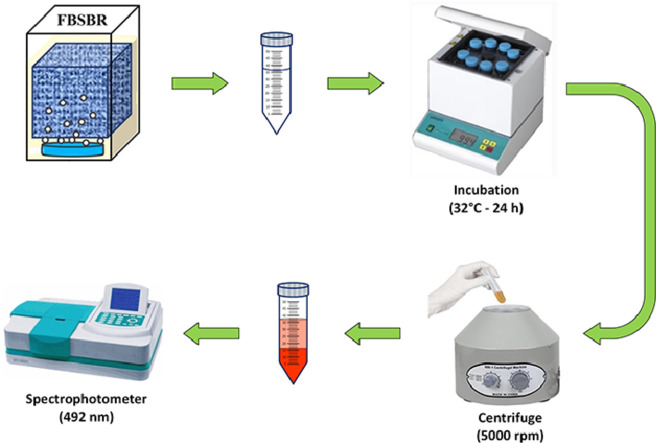

**Table:** Specifications Table

Subject Area	Environmental Science
More specific subject area	Measuring the enzymatic activity of biomass in biological wastewater treatment
Method name	Dehydrogenase Activity
Name and reference of original method	Dehydrogenase Activity has been used for detection of amount of activity of living algae in fresh water [1]. In our study, we have used this method for investigation of DHA in wastewater treating a toxic organic compound which can be an effective method for the investigation of biological wastewater treatment process.
Resource availability	–

## Method details

There are lots of enzymes in the bioreactors dealing with the oxidation of organic compounds [2]. Oxidoradactase enzymes accelerate oxidative-reductive reactions, in other words, they help transfer of electrons from one donor to final electron acceptor. This enzyme group has 6 subgroups: oxygenase, reductase, peroxidase, oxidase, hydroxylase and dehydrogenase [3,4].

**Fig. 1:**
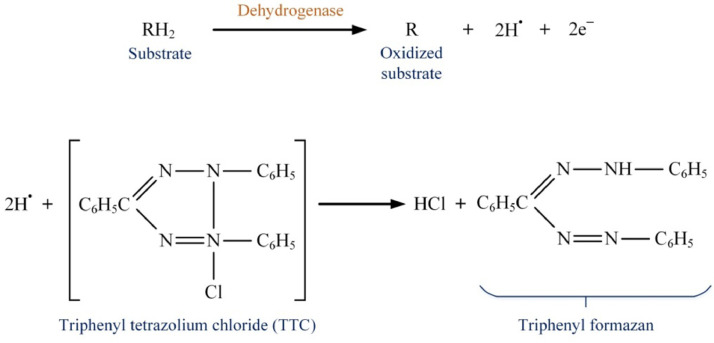
Degradation mechanism of organic substrate by dehydrogenase enzyme.

Intercellular dehydrogenase enzyme is one of the main oxidoreductase enzymes which can be considered as microbial activity indicator. This enzyme plays an important role in the biological oxidation of organic compounds and causes the transfer of hydrogen from the organic substrate to the inorganic acceptor [5]. The mechanism of enzymatic degradation of organic compounds by dehydrogenase enzyme is given in Fig. 1. As shown in the figure, substrate oxidation is reached with a reductive reaction and the removal of one or two hydrogen from the substrate and its transfer to the electron acceptor. The electron acceptors are usually Nicotinamide Adenine Dinucleotide (NAD), Nicotinamide Adenine Dinucleotide Phosphate (NADP), Flavin Adenine Dinucleotide (FAD) and Flavin Mononucleotide (FMN) [1]. Therefore, dehydrogenase enzyme is one of the enzymatic activity if the biomass which is directly affected by the inlet wastewater characteristics. The wastewater characteristics can either stimulate or inhibit the organism's activity. Accordingly, variation of the DHA of microbial content of the bioreactor can be considered as an indicator of the process performance.

**Fig. 2:**
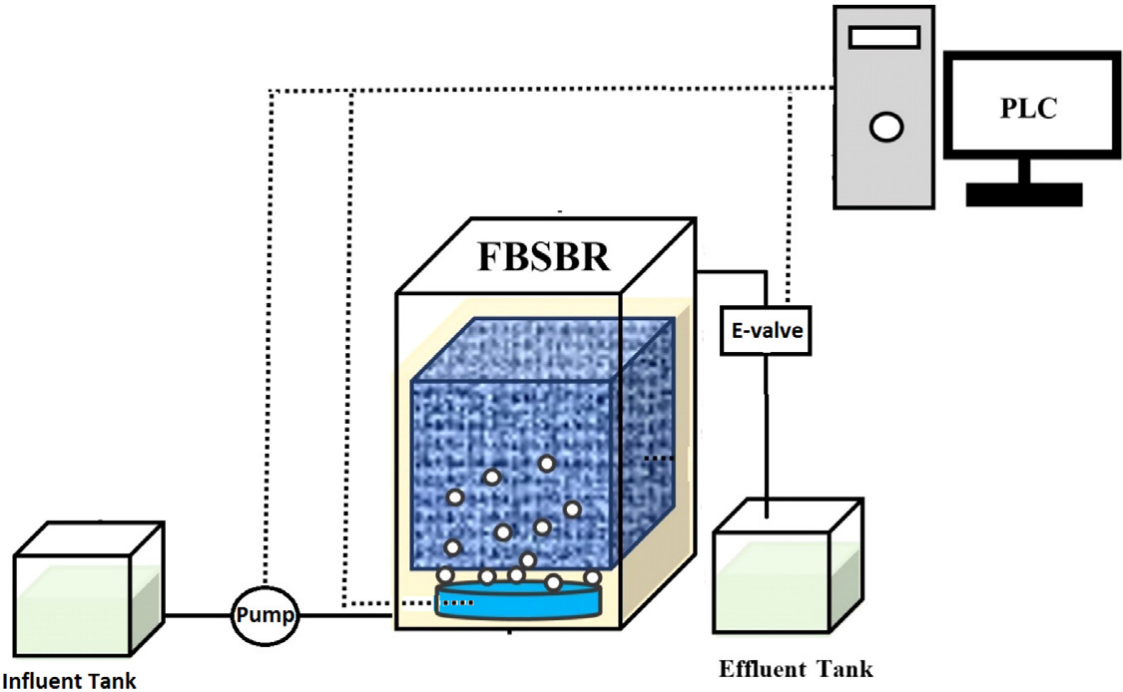
Schematic of bioreactor used for biological degradation of formaldehyde.

Different methods have been reported for measuring DHA in the literature. Strotmann et al. [6] determined the DHA of activated sludge by measuring the reduction of resazurin to resorufin. In another study Feng et al. ([7]) determined the DHA by reduction of 2-(p-iod-phenyl)−3-(p-nitro-phenyl)−5-phenyltetrazolium chloride. Xie et al. [1] used 2,3,5-triphenyl Tetrazoliumchloride (TTC) and Triphenyl Formazan (TF) for measuring the DHA of living algae in fresh water. The methods given in the above-mentioned studies shows that there are variations in sample volume, TTC concentration, incubation time, TF extraction method and solvent, type of substrate and also the TF absorption wavelength. Therefore, the present study aimed to customize the method to make it more concise and simple to measure. The samples for measuring DHA were obtained from a lab scale Fixed Bed Sequencing Batch Reactor (FBSBR) treating formaldehyde. The schematic of the bioreactor is illustrated in Fig. 2. The bioreactor is operated by both attached growth biofilm and suspended growth biomass.

## Chemicals and reagents

For measuring the DHA, TF, TTC, Tris-HCL, Glucose, sodium dithionite, and toluene were used. All chemicals used in the present study were of analytical grade and purchased from Merck Co.

## Experimental procedure

**Fig. 3:**
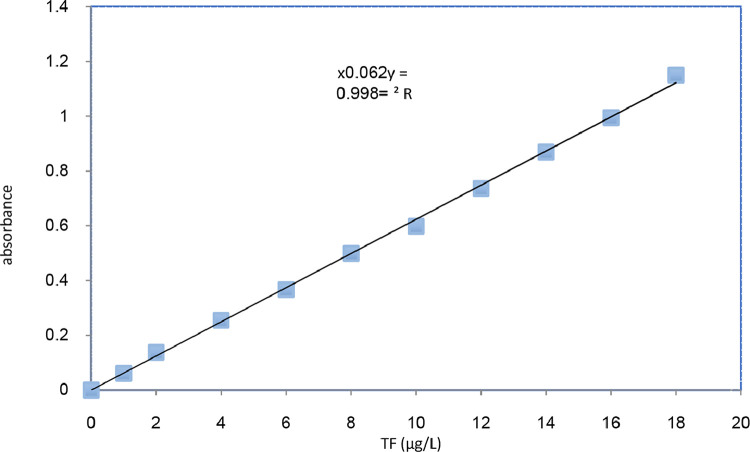
Calibration curve of dehydrogenase activity.

In order to measure the dehydrogenase activity, a calibration curve has been developed using 0.0002 M of TF. The TF solution is made by dissolving TF in toluene. Afterward, the serial TF concentrations of 18, 16, 14, 12, 10, 8, 6, 4, 2 and 1 µg/L were made. The absorption spectra of TF solution revealed that TF has the maximum absorption at 492 nm which is in accordance with similar studies [1]. Then, the calibration curve was developed which is shown in Fig. 3.

**Fig. 4:**
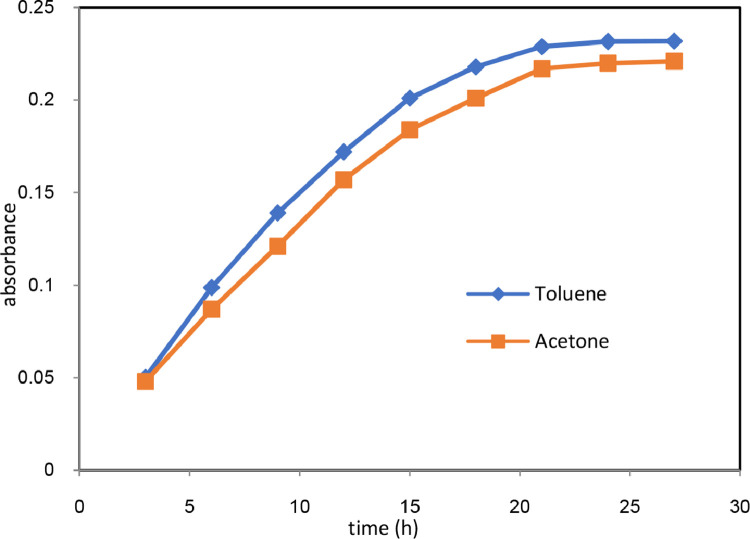
The absorbance of extracted TF at various incubation time and different extraction solvents.

DHA in the bioreactor was calculated based on the Mixed Liquor Suspended Solids (MLSS). Therefore, the samples were taken from bioreactor and MLSS values were measured using the procedure given in standard methods for examination of water and wastewater [8]. In order to measure the DHA, the amount of TF generated by microorganisms in various times was extracted from the samples taken from bioreactor and various incubation times of 3, 6, 9, 12, 15, 18, 21, 24, and 27 h at 37̊C were considered to find the maximum amount of generated TF. In addition, toluene and acetone were used as extraction solvent in the same samples to compare the effect of different solvents for TF extraction. To do this, 10 ml of the mixed liquor was added to a 50 ml falcon tube and 2 mL Tris-HCL with pH of 8.4, 2 mL glucose solution as substrate, and 2 mL of TTC solution each with 0.1 M concentration were added. The falcon tube, then was put in an incubator at 37̊C. After 24 h, the enzymatic activity was stopped by adding sodium dithionate, and then 5 ml of solvents (toluene and acetone) were added to the falcon tube. To extract the generated TF, the content of the tube was centrifuged at 5000 rpm for 10 min. Afterward, the supernatant which was the toluene or acetone solution and the extracted TF was separated and the absorption of this red solution was measured at wavelength of 492 nm. To zero the spectrophotometer, pure toluene and acetone were used. Fig. 4 is illustrating the extracted TF absorbance in various incubation time and solvents. As it is shown in the figure, the majority of the TF is generated in the early times of incubation time and finally it reaches a steady condition after 20 h of incubation. However, the maximum TF absorbance is reached after 24 h of incubation. Therefore, incubation time of 24 h is suggested in this method for TF generation. Furthermore, the results show that the amount of extracted TF in toluene is higher than that of acetone which is proved by the absorbance values at 492 nm. therefore, application of toluene as extraction solvent of TF is preferred to acetone.

Accordingly, the DHA was calculated using the following equations:

(1) $$\text{TF}\;\left(\frac{\mu g\;\text{TF}}{L.d}\right)=\frac{\text{Abs}}{0.0624}$$

(2) $$\text{DHA}\;\left(\frac{\mu g\;\text{TF}}{g_{\text{biomass}}.d}\right)=\frac{\text{TF}}{\text{MLSS}.d}$$

## DHA variation in the bioreactor

In the present study, various parameters were investigated on the performance of the bioreactor to treat formaldehyde. DHA of the biomass was also monitored in the bioreactor when the working condition of the bioreactor was changing. At the start-up section of the study since the microbial contents of the bioreactor were not acclimated with the toxic formaldehyde, DHA activity was 0.35 $\frac{\mu g\;\text{TF}}{g_{\text{biomass}}.d}$, and the COD removal efficiency was as low as 30%. However, at the end of the start-up section, when the complete COD removal efficiency was reached, the DHA was also increased to 0.95 $\frac{\mu g\;\text{TF}}{g_{\text{biomass}}.d}$. Further investigation of other operational parameters such as Hydraulic Loading Rate (HRT) and organic loading rate, showed a considerable reduction in DHA which was along with reduction in COD removal efficiency. However, further operation of the bioreactor at the given condition finally leads to the complete removal efficiency and the corresponding increase in DHA.

## Additional information

Biological wastewater treatment processes are among the acceptable and environmentally friendly ones for degradation of organic contaminants. However, there are lots of operational factors influencing the process performance. On the other hand, the microbial content of the bioreactors are main degrading organisms in the biological processes, and the organic compounds oxidation take place due to the generated enzymes by microorganisms. Therefore, measuring the enzymatic activity of the biological processes could be a promising parameter for the investigation of biological process performance. In the present study, it was found that DHA is directly affected by the organic and hydraulic shocks and maintaining the DHA in an acceptable level could help improve the process performance. In the biological degradation of organic compounds, transfer of TTC to TF gives a chance to measure DHA. There are various parameters in case of DHA measurement. The present study was aimed to investigate the effect of incubation time and type of solvent for the extraction of TF from solution which is greatly influencing the amount of generated and extracted TF. It was found that incubation time of 24 h is the best time for the generation of maximum TF, and extraction of TF into toluene solution is better than that of acetone. Xie et al. [1] have reported incubation time of 90 min for measuring the activity of algae in fresh water which is a short time compared to our study. This could be due to the generation source of TF which is algae. In addition, they have compared the effect of different solvents on extraction of TF. Methanol, ethanol, furan, and acetone were the investigated extractant solvents. They have reported that acetone was the best solution for the extraction of TF. In our study, we compared toluene with acetone as the extractant solvent, and it is found that extraction of TF into toluene is higher than that of acetone. Accordingly, the methods given at this study can be reliable parameter for the better investigation of the suspended biological wastewater treatment process.

## Declaration of Competing Interest

None.
